# Does social influence affect COVID-19 vaccination intention among the unvaccinated?

**DOI:** 10.1017/ehs.2022.29

**Published:** 2022-07-11

**Authors:** Gul Deniz Salali, Mete Sefa Uysal, Gizem Bozyel, Ege Akpinar, Ayca Aksu

**Affiliations:** 1 Department of Anthropology, University College london, 14 Taviton Street, WC1H 0BW, UK; 2 Department of Social Psychology, Friedrich Schiller University Jena, Jena, Germany; 3 Department of Psychology, Dokuz Eylul University, Izmir, Turkey; 4 Deparment of Political Science and International Relations, Altinbas University, Istanbul, Turkey; 5 Department of Psychology, MEF University, Istanbul, Turkey

**Keywords:** Conformism, vaccine hesitancy, psychological reactance, collectivism, cultural evolution

## Abstract

Conformist social influence is a double-edged sword when it comes to vaccine promotion. On the one hand, social influence may increase vaccine uptake by reassuring the hesitant about the safety and effectiveness of the vaccine; on the other hand, people may forgo the cost of vaccination when the majority is already vaccinated – giving rise to a public goods dilemma. Here, we examine whether available information on the percentage of double-vaccinated people affects COVID-19 vaccination intention among unvaccinated people in Turkey. In an online experiment, we divided participants (*n =* 1013) into low, intermediate and high social influence conditions, reflecting the government's vaccine promotion messages. We found that social influence did not predict COVID-19 vaccination intention, but psychological reactance and collectivism did. People with higher reactance (intolerance of others telling one what to do and being sceptical of consensus views) had lower vaccination intention, whilst people with higher collectivism (how much a person considers group benefits over individual success) had higher vaccination intention. Our findings suggest that advertising the percentage of double-vaccinated people is not sufficient to trigger a cascade of others getting themselves vaccinated. Diverse promotion strategies reflecting the heterogeneity of individual attitudes could be more effective.

## Graphical abstract

**Figure:**
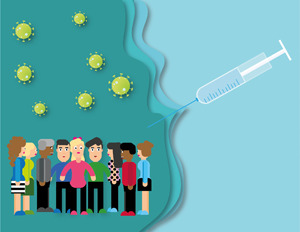

**Social media summary:** Advertising the percentage of vaccinated people is not enough to encourage vaccination among the unvaccinated.

## Introduction

Vaccination is a social dilemma. The more people are vaccinated, the better protected the group is against infectious diseases; however, not everyone is willing to get vaccinated. As vaccination programmes proceed, they can stall because the remaining unvaccinated mainly consist of those who strongly hesitate or refuse to get the vaccine. Ongoing vaccination campaigns provide a natural setting for understanding what strategies work best for increasing vaccine uptake among unvaccinated people. Governments use various strategies to increase vaccination coverage. One of those strategies is using social influence by revealing how many people in the population have received the vaccine and encouraging others to follow. In this study, we tested the effectiveness of this strategy in increasing vaccination intention among the unvaccinated using an online experiment in Turkey.

**Figure 1.**
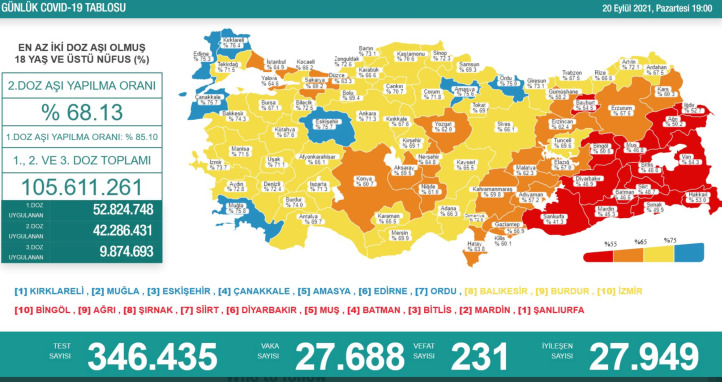
The infographic used by the Turkish Ministry of Health illustrating the percentage of double-vaccinated people in each city at the time of this study in late September 2021. Blue indicates cities where over 75% of the adults have received the two doses of the COVID-19 vaccine, yellow indicates over 65%, orange over 55% and red under 55% (source: https://covid19asi.saglik.gov.tr).

We focused on Turkey for several reasons. First, our previous surveys revealed that general and COVID-19 vaccine hesitancy is high and trust in vaccines low in Turkey compared with other countries (Salali & Uysal, 2020, 2021b). Second, conformist social influence has been actively used as a COVID-19 vaccine promotion strategy in this country. Since late September 2021, the percentage of people who have received at least two doses of the COVID-19 vaccine in each city has been announced daily by the Turkish Health Minister on a colour-coded map on social media, television and the Internet (Figure 1). These announcements included plaudits for the cities that reached over 75% coverage and encouraged others to follow. Third, at the time of our experiment, over 65% of the adult population in Turkey had received the two doses of the COVID-19 vaccine, and the remaining unvaccinated adults in big cities mostly consisted of those who did not get the vaccine despite availability.

### Conformist social influence and the vaccination dilemma

Conformism refers to the tendency of people to learn from and adopt the behaviours of the majority in their group (Asch, 1956). In cultural evolutionary models, conformist transmission (or social influence) is more narrowly defined as an individual bias to *disproportionately* copy the majority, that is, an individual will have a higher probability of acquiring a behaviour than the frequency of that behaviour in the population (Henrich & Boyd, 1998). Evolutionary human scientists have argued that conformism is adaptive unless environments change too rapidly or individual learning is error free (Boyd & Richerson 1985; Henrich & Boyd, 1998). Conformist social influence may contribute to the spread of cultural practices with health consequences. For example, the practice of female genital cutting is found to be frequency dependent – the probability of its occurrence rises with increasing incidence of ethnic female genital cutting (Howard & Gibson, 2017). Social influence may also play a role in the adoption of new health technologies. A new vaccine constitutes a prime example. The novelty raises questions about unforeseen side effects and the efficacy of the vaccine and may lead to hesitancy, especially when the origin of the disease is largely unknown (Salali & Uysal, 2020). Seeing others getting vaccinated may therefore encourage hesitant individuals, especially if they have concerns about vaccine side effects. Through a cross-cultural survey, we found that vaccination of friends and family was amongst the most effective incentives in increasing vaccination intention among participants who were hesitant about getting the COVID-19 vaccine, especially in Turkey (Salali & Uysal, 2021a). Likewise, a recent theoretical model predicted that willingness to be vaccinated against COVID-19 may increase as more and more people get vaccinated (Schmelz & Bowles, 2021). Because conformist social influence depends on the number of people in a community adopting the behaviour, we expect there to be a threshold percentage of people who have already got the vaccine for the vaccination behaviour to take off in a group.

On the other hand, achieving herd immunity through voluntary vaccination represents a public goods dilemma (Bauch & Earn, 2004). People may adopt a ‘cheater’ or ‘free-rider’ strategy to avoid the (perceived) cost of getting vaccinated if most in their community has already received the vaccine (Ibuka et al., 2014). This is especially true for individuals who perceive the cost of vaccination (e.g. unforeseen side effects) to be high (Bauch & Earn, 2004; Fu et al., 2011; Voinson et al., 2015). The temptation to free ride may result in lowered uptake of vaccinations as the percentage of the vaccinated increases. This is because, at higher levels of vaccination coverage, there will be a lower risk of infection for both the vaccinated and the unvaccinated. This scenario represents a classic example of the public goods dilemma (Hardin 1968) and is likely to be responsible for the observed decline in measles vaccine coverage across Europe in the last few decades (Jansen et al., 2003; WHO, 2020).

**Figure 2.**
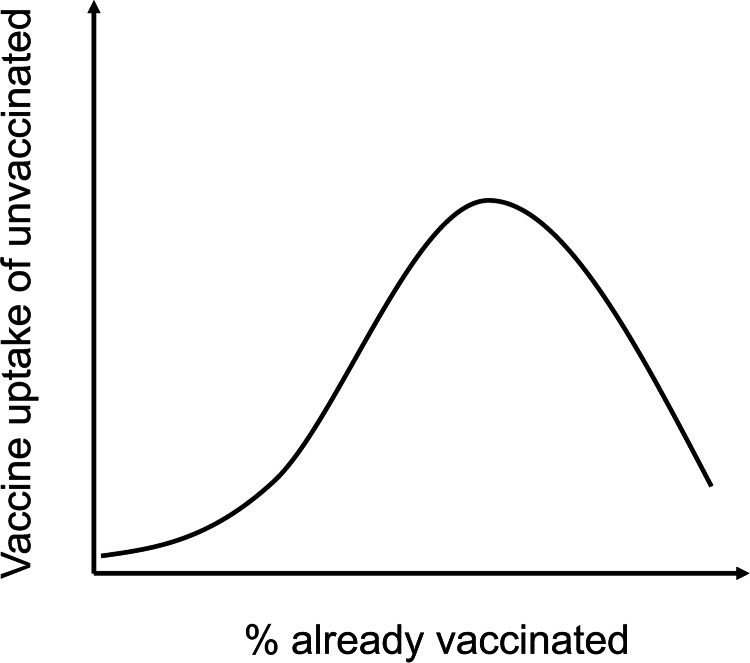
Predicted effect of social influence on the vaccine uptake of unvaccinated people. At lower percentages of vaccine coverage, there will not be enough people for conformist social influence to have an effect. At intermediate percentages, conformist influence will contribute to the increase in vaccination behaviour. At higher percentages, the remaining unvaccinated may free ride on the benefits of herd protection.

Based on the above, we predict that the effect of social influence on vaccine uptake will follow a pattern depicted in Figure 2: at lower percentages of vaccinations in a population, there will not be enough consensus for conformity to kick off. At intermediate levels of vaccination coverage, conformist social influence will amplify vaccine uptake. In standard cultural evolution models for conformist social influence, we observe a sigmoid curve depicting the disproportionate bias in copying the majority (Acerbi et al., 2016). In contrast, in the case of vaccinations, we expect the line to drop down at higher percentages, where the unvaccinated will become disincentivised from vaccinating as they benefit from the growing herd immunity (Figure 2).

### What about the psychology of social influence?

There is another factor that should be considered when examining the effect of social influence on vaccination intention: the susceptibility of an individual to be influenced by the majority (Efferson et al., 2008; Toelch et al., 2013). Psychological attitudes contribute to this susceptibility. For example, individuals who are highly in need of uniqueness are found to resist majority influence (Imhoff & Erb, 2009). Likewise, psychological reactance refers to the defensive response to and intolerance of others telling one how to think (Brehm, 1966) and is negatively correlated with the tendency to conform (Goldsmith et al., 2005). Someone high in reactance perceives advice from others and compliance to social norms as an intrusion on one's freedom and autonomy. Studies have revealed positive links between psychological reactance, belief in conspiracies and vaccine hesitancy (Hornsey et al., 2018) and refusing to wear a facemask during the COVID-19 pandemic (Taylor & Asmundson, 2021). It is possible that psychologically reactant people signal their non-conformity by refusing the COVID-19 vaccine.

Collectivism (the extent to which an individual considers group welfare and loyalty over individual success) may also play a role in vaccine uptake and the degree of the effect of conformist social influence on vaccine uptake. First, a highly collectivistic individual may accept vaccination more readily as they value the benefit of herd immunity. Related research has found that mask usage during the COVID-19 pandemic was higher in more collectivistic US states and countries (Lu et al., 2021). People in more collectivistic (vs. individualistic) cultures may also be more likely to accept various contact-tracing technologies during a pandemic (Arnot et al., 2020). Second, conformist social influence may have a larger influence on collectivistic individuals as they are likely to be more attuned to how people in their group behave. People in more collectivistic cultures, for example, are more likely to conform to majority opinion (Bond & Smith 1996). Likewise, computational models have revealed that majority opinion solidifies more quickly in collectivistic cultures where there is greater susceptibility to social influence (Muthukrishna & Schaller 2020).

Most studies to date have used game theoretical models (Bauch & Earn 2004; Fu et al., 2011; Schmelz & Bowles 2021) or hypothetical disease scenarios (Ibuka et al., 2014) to investigate how social information influences vaccination behaviour. This study uniquely contributes by empirically assessing the role of social influence on vaccination intention among the unvaccinated during the COVID-19 pandemic. It also fills a gap in the literature by examining how psychological factors may mitigate the effect of conformist social influence on the adoption of a new health behaviour. Our study tested the following predictions via an online experiment in Turkey with participants who had not yet been vaccinated against COVID-19:

1. The vaccination intention of unvaccinated individuals will be low at low levels of vaccine coverage, increase with increasing levels and decrease past intermediate levels owing to the vaccination dilemma (Figure 2).
2. Unvaccinated people who score higher on the psychological reactance scale will have lower intention to get the COVID-19 vaccine.
3. Unvaccinated people who score higher on collectivism will have higher intention to get the COVID-19 vaccine.
4. The association with conformist social influence and vaccination intention will be moderated by psychological reactance and collectivism. More specifically, we predict that the positive influence of conformism at intermediate levels of vaccine coverage will not hold for people who score high on reactance. On the other hand, we predict social influence to have a higher effect on people who score higher on collectivism.

## Methods

We conducted an anonymous online experiment with a sample of participants in Turkey from 29 September to 8 October 2021. We used Qualtrics, which is a commercial survey administration company. Qualtrics recruited participants from their pool of potential participants who have agreed to be contacted for the purpose of responding to surveys. Our inclusion criterion was that the participant did not receive any doses of the COVID-19 vaccine (we excluded those who replied ‘yes’ to our initial check question regarding whether they received at least one dose of the vaccine). We used filters to eliminate data from careless or incomplete responses.

In the first part of the online experiment, we gathered information about the psychological and control variables used in the study. Then, we randomly divided the participants into control and experimental conditions and asked manipulation check questions. At the end of the online study, we asked participants about the probability that they would receive the COVID-19 vaccine. The following sections detail each component of the study.

### Psychological and control variables

We measured psychological reactance using the reactance scale (Hong & Faedda, 1996) and collectivism using the collectivism–individualism scale (Hofstede, 2001). As native speakers, we translated all scale questions into Turkish and back-translated them for accuracy. We also gathered information on several demographic variables, including the cities and districts in which the participants were living, as well as sex, age, education level, income and political orientation. We asked about districts to ensure that the participants trusted the information we provided in the experimental condition. Table 1 presents the psychological and demographic variables that were measured in the experiment, the statements employed to capture the variables, the response scales, Cronbach's alphas and means or percentages.

**Table 1.** Summary of the sample and variables

Variable	Statement(s)	Mean (SD)/frequency
Vaccine intention (outcome variable)	What is your probability of getting a COVID-19 vaccine? (0: Definitely will not, 100: Definitely will)	0–25: 31.3%
		26–50: 23.4%
		51–75: 19.8%
		76–100: 25.5%
Psychological reactance (*a =* 0.67)	(1 = completely disagree, 5 = completely agree)	3.59 (0.70)
	1. I become angry when my freedom of choice is restricted	
	2. I find contradicting others stimulating	
	3. It disappoints me to see others submitting to society's standards and rules	
	4. I resist the attempts of others to influence me	
	5. I consider advice from others to be an intrusion	
Collectivism (*a =* 0.89)	(1 = completely disagree, 5 = completely agree)	3.32 (0.88)
	1. Individuals should sacrifice self-interest for the group	
	2. Individuals should stick with the group even through difficulties	
	3. Group welfare is more important than individual rewards	
	4. Group success is more important than individual success	
	5. Individuals should only pursue their goals after considering the welfare of the group	
	6. Group loyalty should be encouraged even if individual goals suffer	
Age		35.95 (9.96)
Gender	Women	67.2%
	Men	32.7%
	Non-binary/other	0.1%
Education level	Elementary school	1.2%
	Middle school	3.1%
	High school	23.9%
	Bachelor's degree	51.3%
	Master's degree	18.1%
	PhD	2.4%
Income level	Less than 2.500 Turkish Liras (TL)	10.1%
	2.500–4.999 TL	21.1%
	5.000–7.499 TL	20.8%
	7.500–9.999 TL	25.8%
	More than 10,000 TL	22.2%
City	İstanbul, Ankara, İzmir	64.6%
	Other cities	35.4%
Political orientation	Left (0–4)	25.7%
	Center (5–6)	45.1%
	Right (7–10)	29.2%

### Experimental conditions

To test our first prediction related to the conformist social influence on vaccination intention, we randomly divided the participants into four experimental conditions. Participants received the following information in each condition: ‘On the next page, you will receive some information. Please read this information carefully before continuing the survey.’

- Control condition: ‘As you know, there has been an ongoing vaccine rollout against the COVID-19 pandemic in our country.’
- Low social influence condition (30% vaccinated): ‘As you know, there has been an ongoing vaccine rollout against the COVID-19 pandemic in our country. As part of this rollout, 30% of the people in the district that you are living in have gotten their two doses of the COVID-19 vaccine.’
- Intermediate social influence condition (60% vaccinated): ‘As you know, there has been an ongoing vaccine rollout against the COVID-19 pandemic in our country. As part of this rollout, 60% of the people in the district that you are living in have gotten their two doses of the COVID-19 vaccine.’
- High social influence condition (90% vaccinated): ‘As you know, there has been an ongoing vaccine rollout against the COVID-19 pandemic in our country. As part of this rollout, 90% of the people in the district that you are living in have gotten their two doses of the COVID-19 vaccine.’

Participants had to wait 7 seconds before moving onto the next page to ensure that they had read the information.

### Manipulation check

To check whether our experimental conditions worked, we asked participants to rate the level of double vaccinations in their district after they received the information assigned to their condition. Specifically, we asked, ‘What do you think about the proportion of people in your district who received the two doses of the COVID-19 vaccine?’ We prompted the participants to choose between the following options: low, intermediate and high. If the experimental manipulation worked, we expected participants in the low social influence condition to choose the ‘low’ option more than those in the control and other conditions. Likewise, we expected participants in the high social influence condition to choose the ‘high’ option more often than those in other conditions. We coded the responses on a scale of 1–3 (1 = low, 2 = intermediate, 3 = high) and compared the responses in the control condition with the others using ANOVA. As predicted, participants in the low social influence condition reported significantly lower scores than those in the control condition (*M*_control_ = 2.04, SD = 0.60; *M*_experiment-low_ = 1.81, SD = 0.66; 95% CI [0.09, 0.39]). Responses in the control and intermediate conditions did not differ (*M*_control_ = 2.04, SD = 0.60; *M*_experiment-intermediate_ = 2.12, SD = 0.57; 95% CI [−0.22, 0.07]). Those in the high social influence condition reported significantly higher scores than those in the control condition (*M*_control_ = 2.04, SD = 0.60; *M*_experiment-high_ = 2.25, SD = 0.70; 95% CI [−0.36, −0.07]). Thus, we concluded that our manipulations had worked as expected.

#### Outcome variable

At the end of the online experiment, each participant was asked to rate their probability of vaccination against COVID-19 on a scale of 0–100 (see Table 1).

### Debriefing

In Turkey, cities are divided into districts. The percentages of people double vaccinated against COVID-19 were provided only at the city level – but not at the district level – by the Turkish Ministry of Health (Figure 1). Given that city-level data were freely verifiable online and may have been known to some participants, we instead chose to share district-level percentages (low/intermediate/high social influence) in our design. This ensured that there was no way for participants to counter check the district-level data they were given against any ‘official’ vaccination rates. For this reason, we added a debriefing statement at the end of the survey informing participants that the shared district-level percentages did not reflect the real percentages of vaccination. We highlighted a link to the official webpage with information on COVID-19 vaccination rates (city by city) and further information on the vaccines (https://covid19asi.saglik.gov.tr).

### Statistical analyses

Because our response variable (vaccination intention) was on a scale of 0–100, we used the logistic curve to describe this data (Crawley, 2012). We corrected for overdispersion by conducting quasibinomial logistic regression analyses in R, using the MASS package (R Development Core Team, 2011; Venables & Ripley 2002). We examined the relationship between (1) social influence conditions (control/30%/60%/90% double vaccinated), (2) psychological reactance score, (3) collectivism score and (4) demographic controls (age, sex, education level, income, political orientation) and COVID-19 vaccination intention (0 [no intention] to 1[full intention]). We used ANOVA to compare the regression models.

## Results

### Social influence did not predict vaccination intention among unvaccinated people

**Figure 3.**
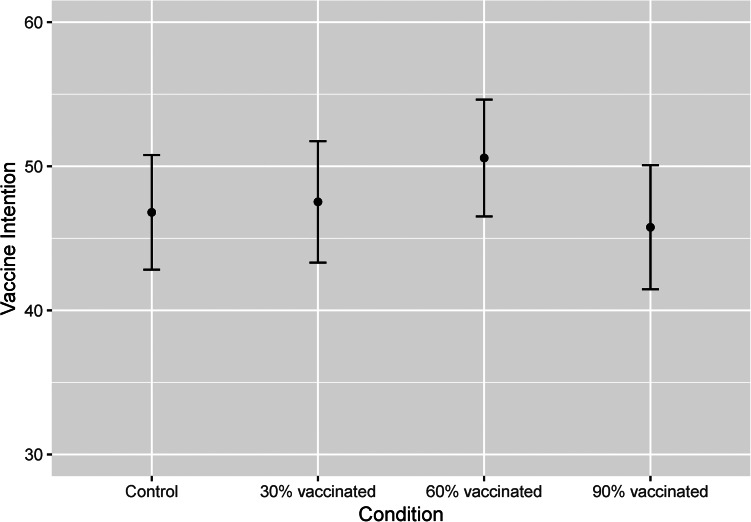
Mean COVID-19 vaccination intention among the unvaccinated in Turkey by experimental conditions (control, *n* = 263; social influence – 30% double vaccinated, *n* = 248; 60% double vaccinated, *n* = 254; 90% double vaccinated *n* = 270). Error bars indicate 95% confidence intervals.

As predicted, the mean vaccination intention was highest among the participants in the intermediate (60% vaccinated) social influence condition (Figure 3). However, contrary to our first prediction, there was no significant difference in the odds of COVID-19 vaccination intention between the control and social influence conditions (Table 2, model 1).

**Table 2.** Logistic regression models of the odds of COVID-19 vaccination intention among the unvaccinated in Turkey

	Model 1		Model 2		Model 3		Model 4	
	Odds ratio (confidence intervals)	*p-*Value	Odds ratio (confidence intervals)	*p-*value	Odds ratio (confidence intervals)	*p-*value	Odds ratio (confidence intervals)	*p-*Value
Low social influence: 30% vaccinated	0.97 (0.77, 1.23)	0.824			0.97 (0.76, 1.23)	0.790	0.34 (0.09, 1.35)	0.125
Intermediate social influence: 60% vaccinated	1.13 (0.90, 1.43)	0.293			1.14 (0.90, 1.44)	0.270	0.48 (0.12, 1.96)	0.310
High social influence: 90% vaccinated	0.94 (0.74, 1.18)	0.579			0.94 (0.74, 1.18)	0.580	0.90 (0.23, 3.51)	0.877
Psychological reactance			**0.80** **(0.71, 0.91)**	0.001	**0.80** **(0.70, 0.91)**	<0.001	**0.81** **(0.64, 1.03)**	0.083
Collectivism			**1.14** **(1.04, 1.27)**	0.009	**1.14** **(1.04, 1.27)**	0.010	0.98 (0.81, 1.19)	0.833
Gender (0 = women, 1 = men)	**0.78** (**0.65, 0.94)**	0.009	**0.78** **(0.65, 0.94)**	0.010	**0.78** **(0.65, 0.94)**	0.010	**0.77** (**0.64, 0.93)**	0.010
Age	1.00 (0.99, 1.01)	0.462	1.00 (0.99, 1.01)	0.861	1.00 (0.99, 1.01)	0.900	1.00 (0.99, 1.01)	0.914
Education	0.93 (0.84, 1.04)	0.189	0.95 (0.86, 1.06)	0.383	0.96 (0.86, 1.06)	0.410	0.96 (0.86, 1.07)	0.418
Income	1.06 (0.98, 1.14)	0.166	1.07 (0.99, 1.15)	0.095	1.07 (0.99, 1.16)	0.080	1.07 (0.99, 1.16)	0.081
Political Orientation (0 = left, 10 = right)	**1.08** (**1.04, 1.11**)	<0.001	**1.07** **(1.03, 1.10)**	<0.001	**1.07** **(1.03, 1.10)**	<0.001	**1.07** (**1.03, 1.10)**	<0.001
Reactance × low social influence							1.11 (0.78, 1.57)	0.560
Reactance × intermediate social influence							0.93 (0.66, 1.32)	0.695
Reactance × high social influence Vaccinated							0.92 (0.65, 1.29)	0.613
Collectivism × low social influence							1.22 (0.92, 1.62)	0.161
Collectivism × intermediate social influence							**1.39** (**1.06, 185)**	0.020
Collectivism × high social influence							1.11 (0.85, 1.46)	0.432

Bold values indicate statistically significant odds ratios.

### Collectivistic unvaccinated had higher, reactant unvaccinated had lower vaccination intention

**Figure 4.**
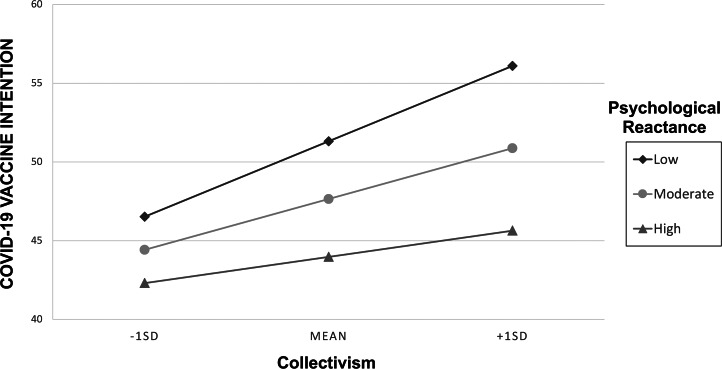
Vaccination intention among unvaccinated people in Turkey by their collectivism and psychological reactance scores.

In line with predictions 2 and 3, both collectivism and psychological reactance scores predicted the odds of vaccination intention among the unvaccinated (Figure 4). The odds of COVID-19 vaccination intention increased by 14% with every one-point increase in the mean collectivism score (Table 2, model 2). In contrast, a point increase in the psychological reactance score was associated with a 25% decrease in the odds of vaccination intention (Table 2, model 2).

One of our predictions (no. 4) was that susceptibility to social influence would be moderated by psychological reactance and collectivism. We expected people who scored higher on psychological reactance to be less influenced by others’ vaccination behaviour. Nevertheless, the reactance × social influence interaction terms were non-significant predictors of vaccination intention (Table 2, model 4). To further test whether social influence changed the odds of vaccination intention among the participants who scored low on reactance, we performed an additional regression analysis with the subgroup of participants whose reactance mean score was <3. Social influence did not predict vaccination intention in this subgroup either (30% condition, *p* = 0.78; 60% condition, *p* = 0.95; 90% condition, *p* = 0.42).

As predicted, there was a positive collectivism × social influence interaction at the intermediate (60%) level (Table 2, model 4). In other words, unvaccinated people who scored higher on collectivism and who received the information that 60% of those in their district were double vaccinated had the highest vaccination intention. Nevertheless, our model testing revealed that the models in which we included experimental conditions (social influence), psychological variables and demographic controls did not differ from the model that only included psychological variables and demographic controls (Table 2, models 2 and 3, *F*(3, 1001) = 1.04, *p* = 0.37; models 2 and 4, *F*(9, 995) = 1.20, *p* = 0.29). Therefore, the minimal adequate model was the one that did not include social influence but only included psychological variables (Table 2, model 2).

Among our control variables, we found that unvaccinated men were 28% less likely to get the COVID-19 vaccine than unvaccinated women (Table 2, model 2). Moreover, a one-point increase from left- to right-wing political orientation was associated with a 7% increase in the odds of vaccination intention (Table 2, model 2). Age, income and education were not significant predictors of COVID-19 vaccination intention among the unvaccinated (Table 2, model 2).

## Discussion

In this paper, we examined whether using the potential of conformist social influence is an effective vaccine promotion strategy. We seized the opportunity of the ongoing COVID-19 vaccine rollout to design a realistic study and captured the attitudes of the remaining unvaccinated. We found that information on the percentage of double-vaccinated people did not influence the odds of COVID-19 vaccination among unvaccinated people in Turkey. Two psychological variables, however, predicted their vaccination intention. People who scored higher on collectivism had higher vaccination intention, and people who scored higher on psychological reactance had lower vaccination intention. Our findings also indicated that, among the remaining unvaccinated, women and those with a right-wing political orientation had higher odds of COVID-19 vaccination.

Using computational models, researchers have demonstrated that the willingness to be vaccinated may increase over time in response to the percentage of the population already vaccinated and argue that the media should focus on reporting those already vaccinated to induce a cascade of vaccinations (Schmelz & Bowles, 2021). Here, we took this a step further and predicted that while this conformist social influence may work at intermediate percentages of vaccination coverage, at high percentages of coverage, social influence may cease to work owing to vaccination becoming a public goods dilemma. Contrary to our prediction, we found no effect of conformist social influence on COVID-19 vaccination intention. It is possible that the available information on the percentage of double-vaccinated people in one's district is not as influential as observing people within one's immediate social network getting vaccinated. For example, in a previous survey study, we discovered that one of the most effective incentives at increasing the reported COVID-19 vaccination intention among vaccine-hesitant people in the UK, US and Turkey was the vaccination of friends and family members (Salali & Uysal, 2021a). An improvement to the current study design would be to ask participants how many of their friends had been vaccinated, and to further test the association between this information and vaccination intention. Moreover, unlike other social coordination problems like mask wearing, vaccination is not a visible trait. Not everyone follows the statistics on vaccination, and one's vaccination status is not visible like a mask. This may also restrict the scope of social influence.

Another possibility is that conformist social influence is not as straightforward as many policymakers hope and there is heterogeneity in individual susceptibility to social influence (Efferson et al., 2020; Toelch et al., 2013). Indeed, our findings suggest that individual-level psychological traits are key. The extent to which a person viewed others’ attempts at persuasion as an intrusion on their freedom (reactance) played a larger role in vaccination intention among the remaining unvaccinated than did social influence. This finding on reactance is consistent with the previous finding that antivaccination attitudes across cultures are strongest among highly reactant people (Hornsey et al., 2018). Moreover, in a demographically representative survey study conducted in March 2021, we found that psychological reactance was higher among Turkish participants than participants in the UK and the US and positively correlated with vaccine hesitancy in all countries (unpublished results). This study included a sample of 1567 participants from Turkey with varying vaccine hesitancy levels. Our further analysis comparing the mean reactance scores of the survey study participants with those of the current study participants revealed a significant difference between the mean scores. Although the distributions of the reactance scores among those two groups were similar (see Supplementary Figure 1), the unvaccinated people in the experimental study had a higher mean reactance score than the mixed pool of participants surveyed in March 2021 (*F*(1, 2580) = 5.07, *p* < 0.05). This difference in the mean reactance scores suggest that those remaining unvaccinated during a vaccine rollout programme probably include more reactant people.

Why do we observe a positive correlation between vaccine hesitancy and psychological reactance? And what does it tell us about conformity? In the same survey study mentioned above, we also found that reactance, belief in health conspiracies, having a general conspiracy mentality and vaccine hesitancy were all positively correlated (unpublished results). One aspect of conspiracy beliefs is that they offer alternative explanations to the majority opinion and attract people with high need for uniqueness who agree less with majorities (Imhoff & Erb, 2009; Imhoff & Lamberty, 2017). The positive links between conspiracy beliefs and the pursuit of uniqueness and conspiracy beliefs and reactance suggest that highly reactant people may refuse to get vaccinated (especially when there is a large vaccine campaign) as a statement of their non-conformity.

While reactant unvaccinated people had lower odds of COVID-19 vaccination, the odds were higher among the more collectivistic unvaccinated. It is possible that people who attach importance to their group's wellbeing consider vaccination even though they hesitate. Moreover, since majority influence is predicted to have a larger effect in collectivistic cultures (Muthukrishna & Schaller, 2020), we expected unvaccinated people high in collectivism to be more affected by social influence. Indeed, we found that at intermediate levels of social influence (60% vaccinated), more collectivistic participants indicated higher vaccination intention. Nevertheless, collectivism and reactance alone, regardless of social influence, were sufficient to explain the variation in the odds of vaccination intention. Therefore, our finding on the collectivism–social influence interaction is inconclusive.

The lack of evidence of social influence that we found here has broader theoretical implications. Cultural evolution models make ample use of conformist transmission, and some conclude that conformism can promote prosocial behaviours like cooperation through cultural group selection (Boyd et al., 2011; Boyd & Richerson, 1982, 1985; Henrich & Boyd, 2001), while others argue that conformism can hinder them (Lehmann et al., 2008; Lehmann & Feldman, 2008; Molleman et al., 2013). Nevertheless, empirical studies testing these models are scarce. Those few empirical studies have findings ranging from no support for conformity (Eriksson & Coultas, 2009), to weak (Claidière et al., 2012) and strong support (Morgan et al., 2012). Given that copying the majority can be adaptive in relatively stable environments (Henrich & Boyd, 1998), we expect conformist transmission not only in humans but also in other animals that rely on social learning. A few empirical studies have shown conformist social influence in primates (Dindo et al., 2009; Whiten et al., 2005), great tits (Aplin et al., 2015) and sticklebacks (Pike & Laland, 2010). However, other researchers have argued that those observations may result from mechanisms other than conformist transmission (Acerbi et al., 2016; Haun et al., 2013; van Leeuwen et al., 2013, 2015). Evolutionary models of conformism also largely ignore individual variation in susceptibility to social influence. A few experimental and empirical studies, however, have illustrated that there is much heterogeneity in social learning strategies (Deffner et al., 2020; Kendal et al., 2018; McElreath et al., 2008) and that not everyone is a conformist or a non-conformist (Efferson et al., 2008, 2015, 2020; Toelch et al., 2013). Future theoretical models should therefore consider individual susceptibility to social influence when employing conformist transmission.

Previous findings have revealed a link between conservative political ideology and antivaccination attitudes (Hornsey, 2020); however, our findings suggest that the direction of this link changes depending on the country. We previously demonstrated that vaccine hesitancy in Turkey decreases, and vaccine trust increases, from left- to right-wing political orientation (Salali & Uysal, 2021b). Here, the odds of vaccination intention among the unvaccinated in Turkey increased with a political orientation towards the right. Another study showed an increased likelihood of COVID-19 vaccination among the politically right wing in Mexico but a decreased likelihood among those in the US (Roozenbeek et al., 2020). The link between political orientation and vaccine hesitancy probably comes down to trust in government and specific institutions (Jamison et al., 2019). It is possible that governments’ COVID-19 vaccine promotion campaigns and messages (simulated here) are taken more sympathetically among the right wing, who have greater trust in the government than the left wing. The lack of trust in the government among the unvaccinated left wing may contribute to their observed vaccination intention. Further studies should test the link between trust in government and vaccination decisions in Turkey.

### Policy implications

Applications of cultural evolution theory to public policy for behaviour change can be fruitful (Muthukrishna, 2020). Our findings add to this growing endeavour and suggest that advertising the percentage of those who have already received two doses of the COVID-19 vaccine in Turkey is not sufficient to induce a cascade of others getting themselves vaccinated. What is the take-home message for a policymaker, especially given the association of attitudes like reactance and collectivism with vaccination intention? There is clearly no ‘one-size-fits-all’ strategy given the heterogeneity of attitudes associated with vaccine hesitancy. Policymakers should have a solid understanding of their society's traits, like collectivism, and deploy different strategies accordingly (Jarrett et al., 2015; Schimmelpfennig et al., 2021; Simas & Larson, 2021). In Turkey, the use of statistics on the percentage of double vaccinated may have no traction, but emphasising the group benefit (i.e. ‘get your vaccine and protect your local community’) may be a better nudge for vaccine-hesitant people who are more collectivistic. The same message, however, may not induce any change in reactant people. While some researchers suggest that vaccine mandates will induce more reactance and result in unsuccessful vaccine promotion (Schmelz & Bowles, 2021), others argue that the mandates will not necessarily backfire and can lead to greater compliance (Albarracin et al., 2021). Especially for highly reactant people, more creative and less controlling communication strategies emphasising individual freedom should be considered (Hornsey, 2020; Miller et al., 2007). With the right incentives, people may act beyond their self-interest (Chapman et al., 2012).

### Limitations and future directions

Like most experimental studies conducted through the Internet, our sample had some limitations. For example, 520 out of 1013 participants had an undergraduate degree – a relatively high percentage compared with country-specific education levels. Nevertheless, we controlled for education level, which did not emerge as a significant predictor of COVID-19 vaccination intention in our models. Furthermore, 65% of our participants came from the three most populated cities in Turkey. Therefore, the generalisability of our findings to under-represented cities and regions is not clear. Moreover, because our participation criterion was no prior doses of the COVID-19 vaccine, our sample could not be demographically representative. As such, there was a bias towards female participants (Table 1). For this reason, the gender differences found in this study should be interpreted carefully. Our result suggests that *among those who had not yet received the COVID-19 vaccine*, women's vaccination intention was higher than men's.

Our design used deception as the rates of vaccinations in the experimental conditions did not reflect the actual rates. In fact, neither the experimenters nor the participants knew the district-level vaccination rates as these were not available publicly. Our study questions necessitate this design as we would not be able to introduce the variation in vaccination rates without the use of deception. To avoid negative consequences that might arise owing to deception, we debriefed participants at the end of the study and provided an official link to the city-level vaccination rates. The use of deception in experimental psychology is common and studies have found that adequate debriefing limits the potential negative effects to participants (Boynton et al., 2013; Smith & Richardson, 1983).

We also acknowledge that vaccination intention is not a perfect proxy for actual uptake. A few studies have investigated the correlation between vaccination intention and uptake. A study conducted in the Netherlands found that the intention to get vaccinated against influenza was a good predictor of vaccine uptake among healthcare workers (Lehmann et al., 2014). Likewise, a longitudinal study in China found that higher vaccination intention predicted actual COVID-19 vaccine uptake (Wang et al., 2022). Nevertheless, future studies would benefit from a longitudinal design, where follow-up questions reveal whether unvaccinated people ultimately received the vaccine or not in the following months. Our study concerned the unvaccinated in Turkey; future studies should test whether conformist social influence affects the decision of the unvaccinated in other cultures.

## Conclusion

In sum, our results suggest that conformist social influence does not change the odds of COVID-19 vaccination intention among unvaccinated people in Turkey. Instead, they offer new insight into the psychological factors that contribute to vaccination decisions. Understanding these factors facilitates the development of more effective vaccine promotion strategies.

## Research transparency and reproducibility

All data files and the R code are available at https://osf.io/jb7zs/?view_only=bfd3cb07ee3d478297bb55a9d19b659b

## Supplementary material

For supplementary material accompanying this paper visit https://doi.org/10.1017/ehs.2022.29. click here to view supplementary material
