# Supplementary material for: Does social influence affect COVID-19 vaccination intention among the unvaccinated?
Source: Evol Hum Sci. 2022 Jul 11;4:e32. doi: 10.1017/ehs.2022.29 (PMC10426110; doi:10.1017/ehs.2022.29)
Supplement: Supplementary file 1 [file S2513843X22000299sup001.docx]

**Does social influence affect COVID-19 vaccination intention among the unvaccinated?**

Gul Deniz Salali^1*^, Mete Sefa Uysal^2^, Gizem Bozyel^3^, Ege Akpinar^4^, Ayca Aksu^5^

^1^Department of Anthropology, University College London, 14 Taviton Street, WC1H 0BW, United Kingdom

^2^Department of Social Psychology, Friedrich Schiller University Jena, Jena, Germany

^3^Department of Psychology, Dokuz Eylul University, Izmir, Turkey

^4^Deparment of Political Science and International Relations, Altinbas University, Istanbul, Turkey

^5^Department of Psychology, MEF University, Istanbul, Turkey

*Correspondence: guldeniz.salali@ucl.ac.uk

Supplementary Figure 1. The distribution of reactance scores of participants from the March 2021 survey study (n= 1567, demographically representative sample) and the experimental study conducted in September 2021 (n= 1013, sample only including participants with no prior COVID-19 vaccination).
